# Effectivity of homecare and professional biofilm removal procedures on initial supragingival biofilm on laser-microtextured implant surfaces in an ex vivo model

**DOI:** 10.1186/s40729-021-00326-x

**Published:** 2021-05-21

**Authors:** Gordon John, Frank Schwarz, Alexandra Kravchenko, Michelle Alicia Ommerborn, Jürgen Becker

**Affiliations:** 1 Department of Oral Surgery and Central Admittance, Westdeutsche Kieferklinik, Heinrich Heine University Düsseldorf, Moorenstr. 5, D-40225 Düsseldorf, Germany; 2 Department of Oral Surgery and Implantology, Carolinum, Goethe University, Frankfurt, Germany; 3 Department of Operative Dentistry, Periodontology and Endodontology, Heinrich Heine University, Düsseldorf, Germany

**Keywords:** Peri-implantitis, Implant surfaces, Implant decontamination

## Abstract

**Background:**

The aim of the current study was the evaluation of initial biofilm adhesion and development on laser-microtextured implant collar surfaces and the examination of effectivity of different biofilm management methods.

**Methods:**

Initial biofilm formation was investigated on hydrophobic machined and laser-microtextured (Laser-Lok) titanium surfaces and hydrophobic machined and laser-microtextured (Laser-Lok) titanium aluminium vanadium surfaces and compared to hydrophobic smooth pickled titanium surfaces, hydrophilic smooth and acid etched titanium surfaces, hydrophobic sandblasted large grid and acid etched titanium surfaces (titanium Promote) via erythrosine staining and subsequent histomorphometrical analysis and scanning electron microscopic investigations. After decontamination procedures, performed via tooth brushing and glycine powder blasting, clean implant surface was detected via histomorphometrical analysis.

**Results:**

After 24 h mean initial plaque area was detected in the following descending order: smooth pickled titanium > titanium Promote > hydrophilic smooth and acid etched titanium > Laser-Lok titanium > Laser-Lok titanium aluminium vanadium. The same order was determined after 48 h of biofilm formation. After glycine powder blasting all samples depicted almost 100% clean implant surface. After tooth brushing, Laser-Lok titanium (67.19%) and Laser-Lok titanium aluminium vanadium (69.80%) showed significantly more clean implant surface than the other structured surfaces, hydrophilic smooth and acid etched titanium (50.34%) and titanium Promote (33.89%). Smooth pickled titanium showed almost complete clean implant surface (98.84%) after tooth brushing.

**Conclusions:**

Both Laser-Lok surfaces showed less initial biofilm formation after 24 and 48 h than the other implant surfaces. In combination with the significant higher clean implant surfaces after domestic decontamination procedure via tooth brushing, both Laser-Lok surfaces could be a candidate for modified implant and abutment designs, especially in transmucosal areas.

## Introduction

Already in 2008, it was indicated that in up to 80% of the patients and in up to 50% of dental implants, peri-implant mucositis occurs. Regarding peri-implantitis, prevalence was described up to 56% of the patients and up to 43% of the implants [1]. Peri-implant infections are mainly caused by bacterial attachment to the implant surfaces and subsequently the development of a biofilm [2]. Bacterial colonization and biofilm development begin immediately after exposure of the implant surface to the oral cavity, when the implant surface is covered by an initial pellicle layer, which consists of organic elements [3, 4]. Without any curative treatment, this condition can lead to peri-implant mucositis, further to peri-implantitis and can even result in implant loss [5]. For that reason, it would be desirable to use implant surfaces exhibiting lower adherences to bacterial organisms and prevent or decelerate biofilm development, especially for the vulnerable transmucosal section. It is claimed that laser-microtextured implant collar surfaces, Laser-Lok (LL) surfaces, could have an advantage regarding a physiological sealing of this vulnerable implant collar section [6]. Also in clinical investigations promising results could be monitored. It was indicated that Laser-Lok surfaces showed less initial crestal bone loss compared to implants with non Laser-Lok surfaces [7]. Generally, regarding initial bone stability after inserting dental implants, good outcomes could be detected [8–10]. The aspect of bacterial adhesion and biofilm development has not been investigated yet, although it is also an important issue for implant maintenance.

The purpose of the current study was the evaluation of initial natural biofilm development on Laser-Lok implant surfaces in comparison to different established implant surfaces. Another important question is the effectivities of domestic hygiene procedures like tooth brushing and professional biofilm management treatments like air abrasive device with glycine powder, which were also evaluated in the current study.

## Materials and methods

### Study population

The design and protocol of the current study was approved by the ethical committee of the University of Düsseldorf. In the current study 5 volunteers were included (2 women, 3 men, mean age 28.0 ± 4.5). A detailed description of the study was given to each subject. Prior to the start of the study every proband had to sign an informed consent. The following inclusion criteria were set: (1) good level of oral hygiene (PI < ), (2) no signs of inflammation of the surrounding soft tissues, (3) no antibiotic therapy during the last six months, and (4) nonsmokers. Probands being younger than 18 years or being older than 60 years were excluded. Probands of both sexes were allowed to take part in the study. They were introduced to maintain their dietary habits and to wear the splint all day. For the time of toothbrushing, the volunteers were allowed to remove the splint from the oral cavity. They were also allowed to brush the splint carefully on the teeth covering parts of the splint, without contact to the samples.

### Intraoral splints and titanium discs

**Fig. 1:**
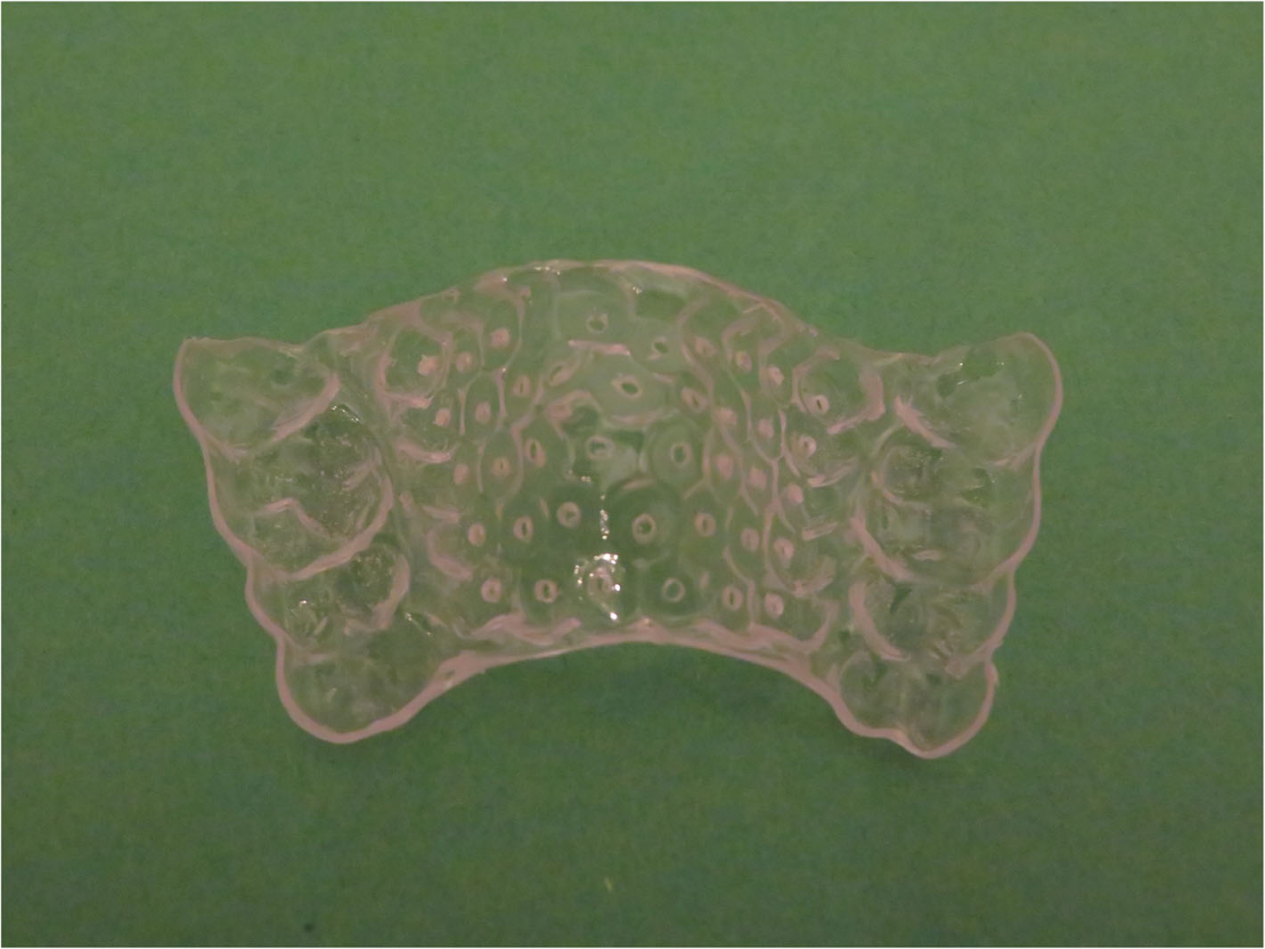
Illustration of an intraoral splint

Samples, in diameter of 5 mm and 2 mm thickness, were made of titanium grade 4 with 4 different surfaces and titanium aluminium vanadium (Ti-6Al-V4 ELI) with 1 surface and were inserted in special splints and protected against dislocation with cyanoacrylate glue (Loctide 496, Henkel AG & Co. KGaA, Düsseldorf, Germany), a hydrophobic machined commercially pure titanium surface (spCPTi, smooth pickled CPTi, Rz: 11.42 μm, Ra: 2.22 μm), a hydrophilic modified smooth and acid etched titanium surface (sehCPTi, smooth etched hydrophilic CPTi, Rz: 1.9 μm, Ra: 0.34 μm), a hydrophobic sandblasted large grid and acid etched titanium surface (CPTi Promote, Rz: 6.06 μm , Ra: 1.02 μm), a hydrophobic machined and laser-microtextured titanium surface (LL CPTi, CPTi Laser-Lok, groove width of 8.1 μm, groove depth of 8.7 μm), and a hydrophobic machined and laser-microtextured titanium aluminium vandium surface (LL TAV, TAV ELI Laser-Lok, groove width of 8.0 μm, groove depth of 9.1 μm). All samples were provided by CAMLOG Biotechnologies AG, Basel, Switzerland, and allocated randomly to the splints by the use of a computer-generated list (Randlist, DatInf GmbH, Tübingen, Germany). The splints were worn in the upper jaw for the period of biofilm formation of 48 h. The design of the splints (Fig. 1) was chosen that the discs were turned to the palate in a distance of 1 mm for providing a moist and nutritious environment. During time of biofilm formation, probands were instructed to maintain their regular diet as well as to keep the splints intraorally, except during the time for tooth brushing. Only mechanical tooth brushing with water was permitted during these plaque accumulation times of 24 h or 48 h without the use of tooth paste or mouth rinsing solutions.

### Detection of initial plaque area (IPA)

For investigation of initial biofilm formation, the timeframe of 24 and 48 h was determined. Immediately after biofilm formation, the splints were removed from the proband’s mouth, and the samples were removed from the splints, carefully rinsed with water, and stained with erythrosine (Erythrosine B, Certistain; Merck KGaA, Darmstadt, Germany). Subsequently, ten samples of each group were photographed at a magnification of eight by the use of a stereo microscope (SZ61; Olympus Europa Holding GmbH, Hamburg, Germany) and a digital camera (ColorViewIII; Olympus Holding GmbH, Hamburg, Germany). For quantitative analysis of initial plaque area, histomorphometrical analysis was performed by the use of a professional analysing and documentation software (Cell D, Olympus Europa GmbH, Hamburg, Germany). Measurements were taken in square fields at the size of 4 mm^2^. Ten of these fields per sample were placed at random and investigated. The coverage of the sample surface by initial plaque area was measured in percentage. The investigation was done by an experienced examiner, being masked to the study conditions.

### Cleansing procedure and detection of clean implant surface (CIS) after treatment procedures

For the investigation of cleansing procedures, only samples with 48 h biofilm formation were used. To imitate the situation of patients mechanical cleansing of the implants, a toothbrush (Meridol® toothbrush, GABA GmbH, Lörrach, Germany) was used to clean the sample surfaces. This toothbrush is featured by multiple, nearly 1700 soft fibres, being very tenuous. After erythrosine dyeing and photographing, the samples were cleaned by circumduction of the toothbrush until no biofilm could be removed macroscopically. For every sample, a new toothbrush was used. For imitation of professional decontamination procedure, an air abrasive device (Air-Flow Master®, E.M.S. Electro Medical Systems GmbH, Munich, Germany) was used with glycine powder (Air-Flow® Perio Powder, E.M.S. Electro Medical Systems GmbH, Munich, Germany). The blasting procedure was performed with 90° angle of application, a static water pressure of 4.5 bar and a static air pressure of 6 bar in a distance to the sample surface of 2 mm. Decontamination procedures were performed by an experienced dentist. The time needed for cleansing procedures were also taken as a parameter. Treatment time was defined as the time needed until macroscopically no biofilm could be removed. After the cleansing procedure the samples, eight per group, were again photographed and a second histomorphometrical analysis was performed accordingly to the procedure regarding initial plaque area determination to detect clean implant surface as a control of the effectiveness of the brushing on the different implant surfaces.

### Scanning electron microscopy

Immediately after biofilm formation period, two samples per 24 h group were gently rinsed with pure water, dehydrated in increasing concentrations of acetone (40 to 100% in steps of 10%). After drying in hexamethyldisilazane, samples were sputtered with gold and examined using SEM (S-3000 N; Hitachi, Pleasanton, CA, USA). The surface morphology was descriptively evaluated by one experienced examiner being masked to the particular conditions of the study.

### Cell culture

To evaluate the biocompatibility of the biofilm-coated surfaces, 6 samples per group were chosen and used for cell viability measurement. Directly after the performance of cleansing procedures, samples were autoclaved and settled with Human Gingival Fibroblasts (HGF, pass: 5, Provitro GmbH, Berlin, Germany). Additionally, 6 native specimens with smooth pickled titanium, hydrophilic smooth and acid etched titanium, titanium Promote, Laser-Lok titanium and Laser-Lok titanium aluminium vanadium surfaces were taken as control groups. Ten thousand human gingival fibroblast cells per sample were cultured in 1 ml of Dulbecco’s modified Eagle’s medium (DMEM high glucose, Glutamax; Sigma-Aldrich, Schnelldorf, Germany) with the supplement of 10% foetal bovine serum (FBS, Sigma-Aldrich, Schnelldorf, Germany) and 1% penicillin/streptomycin per well in non-binding 24-well plates (Corning® Ultra-low attachment 24-well plate, Sigma-Aldrich, Schnelldorf, Germany). The cell culture conditions were set at a temperature of 37 °C, a humified atmosphere of 95% and 5% CO_2_. The change of nutrition medium was performed after three days. Cell viability was measured on day six by the use of a luminescence assay (CellTiter-Glo®, Promega, Mannheim, Germany) in a luminometer (Victor X3, PerkinElmer, Rodgau, Germany). The signal was measured in counts per second (CPS).

### Electron dispersive X-ray analysis

Two samples of native, unworn smooth pickled titanium, hydrophilic smooth and acid etched titanium surface, titanium Promote, Laser-Lok titanium, Laser-Lok titanium aluminium vanadium surfaces and the corresponding surfaces with 48 h biofilm collecting period were subjected to critical scrutiny via Energy-dispersive X-ray spectroscopy (S-3000 N, Hitachi, Pleaston, USA). Compositions of the surface materials were detected and elements were stated in weight percentage.

### Statistical analysis

Statistical analysis was performed using professional analysing software (SPSS 25, IBM Deutschland GmbH, Ehningen, Germany). Medians, mean values and standard deviations were calculated for each group. Normal distribution was tested via Shapiro-Wilk testing. Differences in initial plaque area and clean implant surface were investigated using Kruskal-Wallis testing. For detecting significant differences between the groups regarding the cleansing time, analysis of variance was performed with post hoc testing using Tamhane T2. Differences in cell viability were detected via non-parametric testing using Kruskal-Wallis testing. Results were considered to be statistically significant at a level of *p* < 0.05%.

## Results

### Initial plaque areas

Mean initial plaque areas with corresponding standard deviations as well as medians are listed in Table 1. Regarding the 24-h groups, no significant differences could be detected between smooth pickled titanium, hydrophilic smooth and acid etched titanium and titanium Promote surfaces, *p* > 0.05. Laser-Lok titanium aluminium vanadium surface showed the lowest initial plaque area, being comparable to Laser-Lok titanium surface, *p* > 0.05. Both groups showed a significant lower initial plaque area than smooth pickled titanium, hydrophilic smooth and acid etched titanium and titanium Promote, *p* < 0.05. Similar tendencies could be observed within the 48-h groups. Smooth pickled titanium, hydrophilic smooth and acid etched titanium and titanium Promote surfaces showed no significant differences, *p* > 0.05. Laser-Lok titanium and Laser-Lok titanium aluminium vanadium also showed comparable results, *p* > 0.05. Both surfaces showed significant less initial plaque area than the three groups mentioned above *p* < 0.05.

**Table 1:** Overview on mean initial plaque area ± standard deviations as well as medians regarding the groups

Implant surfaces	spCPTi	sehCPTi	CPTi Promote	LL CPTi	LL TAV
**24 h**					
Mean in %	92.57	87.36	90.94	58.20	42.84
± SD	5.2	5.0	4.6	11.9	14.3
Median in %	93.51	88.95	90.25	59.08	39.84
**48 h**					
Mean in %	99.75	97.25	99.34	83.37	80.08
± SD	0.6	2.0	0.9	7.7	7.3
Median in %	99.97	98.08	99.67	83.75	78.54

*spCPTi* smooth pickled titanium, *sehCPTi* hydrophilic smooth and acid etched titanium, *CPTi Promote* titanium Promote, *LL CPTi* Laser-Lok titanium, *LL TAV* Laser-Lok titanium aluminium vanadium

### Clean implant surface after decontamination procedures

Results regarding clean implant surface after cleansing procedures are listed in Table 2. Decontamination procedure via air abrasive device resulted in almost complete clean surfaces on all tested surfaces. Clean implant surface could be detected in the following descending order:

**Table 2:** Overview on mean clean implant surface ± standard deviations as well as medians after decontamination procedures (after 48 h biofilm formation) regarding the groups

Implant surfaces	spCPTi	sehCPTi	CPTi Promote	LL CPTi	LL TAV
**Air abrasive device**					
Mean in %	99.91	99.78	99.71	99.94	99.82
± SD	0.1	0.4	0.3	0.1	0.1
Median in %	99.92	99.93	99.76	99.95	99.84
**Toothbrush**					
Mean in %	98.84	50.34	33.89	67.19	69.80
± SD	2.0	7.5	8.7	7.7	7.6
Median in %	99.42	50.22	35.82	67.55	70.17

*spCPTi* smooth pickled titanium, *sehCPTi* hydrophilic smooth and acid etched titanium, *CPTi Promote* titanium Promote, *LL CPTi* Laser-Lok titanium, *LL TAV* Laser-Lok titanium aluminium vanadium

Laser-Lok titanium > smooth pickled titanium > Laser-Lok titanium aluminium vanadium > hydrophilic smooth and acid etched titanium > titanium Promote.

Promote surface showed significant less clean implant surface than Laser-Lok titanium, smooth pickled titanium and Laser-Lok titanium aluminium vanadium, *p* < 0.05. The other groups showed similar results, *p* > 0.05.

The toothbrush groups showed a significantly less clean implant surface than all groups, treated with air abrasive device, *p* > 0.05. Clean implant surface of the toothbrush groups could be determined in the following descending order:

smooth pickled titanium > Laser-Lok titanium aluminium vanadium > Laser-Lok titanium > hydrophilic smooth and acid etched titanium > titanium Promote.

Smooth pickled titanium surface showed a significantly higher clean implant surface than all other toothbrush groups, *p* > 0.05. Between Laser-Lok titanium aluminium vanadium and Laser-Lok titanium surfaces, no significant differences could be determined, *p* > 0.05. Both surfaces depicted a significantly higher clean implant surface after toothbrushing than hydrophilic smooth and acid etched titanium and titanium Promote surfaces, *p* < 0.05. The difference between both latter surfaces was also significant, *p* < 0.05.

### Treatment time

**Fig. 2:**
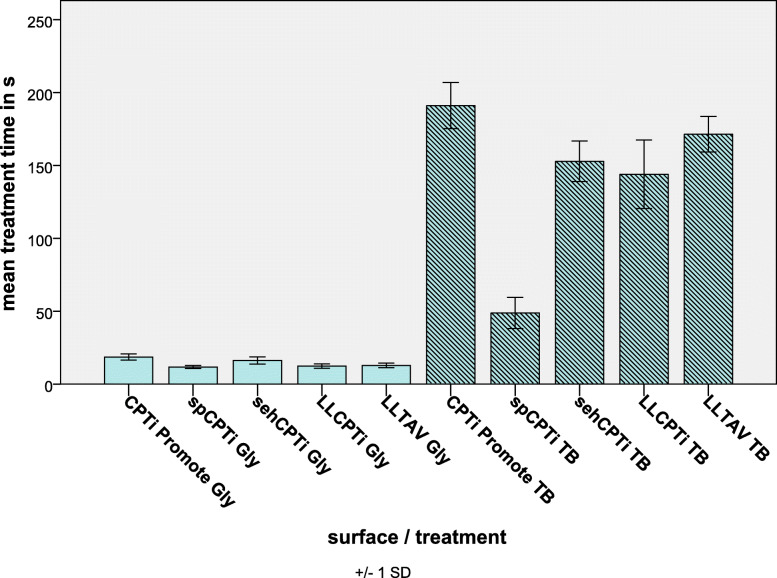
Overview of treatment times, required to remove biofilm macroscopically via toothbrushing (TB) or air abrasive device (Gly) regarding the groups. spCPTi, smooth pickled titanium; sehCPTi, hydrophilic smooth and acid-etched titanium; CPTi Promote, titanium Promote; LL CPTi, Laser-Lok titanium; LL TAV, Laser-Lok titanium aluminium vanadium

All air abrasive device groups showed significant shorter treatment times than all toothbrush groups, *p* < 0.05. The mean treatment time within the air abrasive device groups was determined and ranked in descending order: titanium Promote (18.6 s ± 2.1 s) > hydrophilic smooth and acid-etched titanium (16.3 s ± 2.5 s) > Laser-Lok titanium aluminium vanadium (12.9 s ± 1.6 s) > Laser-Lok titanium (12.4 s ± 1.5 s) > smooth pickled titanium (11.8 s ± 1.0 s) (Fig. 2). The processing time measured in the smooth pickled titanium group was significantly lower than the treatment time of the Promote and hydrophilic smooth and acid-etched titanium groups, *p* < 0.05, respectively. The Laser-Lok titanium and Laser-Lok titanium aluminium vanadium groups depicted significant shorter treatment times than the Promote group, *p* < 0.05, respectively. The mean processing time within the toothbrush groups was ascertained in the following descending order: Promote (191.1 s ± 15.9 s) > Laser-Lok titanium aluminium vanadium (171.5 s ± 12.2 s) > hydrophilic smooth and acid-etched titanium (152.9 s ± 13.9 s) > Laser-Lok titanium (144.0 s ± 23.5 s) > smooth pickled titanium (48.9 s ± 10.7 s) (Fig. 2). The mean treatment time of the smooth pickled titanium group was significantly lower than that of all other groups, *p* < 0.001. The mean treatment time of the hydrophilic smooth and acid-etched titanium and Laser-Lok titanium was significantly lower than the Promote group, *p* < 0.05.

### Cell culture

During the whole study period, no signs of bacterial or fungal contamination could be detected. Results of cell viability after six days of incubation with human gingival fibroblasts are listed in Table 3. Cell viability on the unworn, native control samples was significantly higher than on all biofilm settled and decontaminated samples, *p* < 0.05. Between the sole control groups, no significant differences could be observed as well as between the sole decontaminated groups, *p* > 0.05, respectively.

**Table 3:** Overview on cell viability in mean counts per second (CPS) with corresponding standard deviations as well as medians regarding the groups

Implant surfaces	spCPTi	sehCPTi	CPTi Promote	LL CPTi	LL TAV
**Native samples**					
Mean in CPS	151,065.00	154,388.67	71,099.17	211,414.00	230,479.17
± SD	48919.0	36078.8	15772.0	31,274.3	33,512.6
Median in CPS	148,657.50	136,548.50	62,266.00	220,817.50	238,739.00
**Air abrasive device using glycine powder**					
Mean in CPS	156.33	36.67	39.50	114.00	104.33
± SD	67.6	10.6	6.8	15.6	45.5
Median in CPS	161.50	37.00	40.50	114.00	104.00
**Toothbrush**					
Mean in CPS	141.67	53.83	50.17	111.00	105.33
± SD	78.6	24.4	17.9	80.8	55.4
Median in CPS	140.50	52.00	49.00	82.00	100.00

*spCPTi* smooth pickled titanium, *sehCPTi* hydrophilic smooth and acid-etched titanium, CPTi *Promote* titanium Promote, *LL CPTi* Laser-Lok titanium, *LL TAV* Laser-Lok titanium aluminium vanadium

### EDX analysis

As expected, the highest percentages of titanium could be observed in the unworn, native groups. In these unworn groups, oxygen could be detected in all groups, instead of the hydrophilic smooth and acid-etched titanium group, in which sodium and chloride could be found. Both elements are remnants of the sodium chloride solution in which the samples are stored. In both the Laser-Lok groups, additionally, nitrogen could be observed. In the native Laser-Lok titanium aluminium vanadium group also aluminium as well as vanadium, both being alloying elements in this surface material, could be detected. In the biofilm settled and treated groups higher percentages of nitrogen and carbon could be observed. These percentages were higher in the toothbrush group than in the air abrasive device group, being comparable to the higher residual plaque areas on the toothbrush treated samples (Table 4).

**Table 4:** Overview on mean weight percentages of chemical elements of the different implant surfaces without and after treatment procedures regarding the groups

Implant surfaces	spCPTi	sehCPTi	CPTi Promote	LL CPTi	LL TAV
**Native samples**					
Mean Ti %	90.14	75.68	91.19	88.11	81.00
Mean O%	9.86		8.81	8.41	8,16
Mean Na %		13.76			
Mean Cl %		10.56			
Mean N %				3.48	1.90
Mean Al %					6.73
Mean V %					2.21
**Air abrasive device using glycine powder**					
Mean Ti %	64.11	65.56	67.82	53.66	60.62
Mean O %	10.72	10.80	9.30	14.65	11.00
Mean C %	11.65	6.18	8.12	15.57	7.24
Mean N %	13.52	17.46	14.76	16.12	16.29
Mean Al %					4.85
**Toothbrush**					
Mean Ti %	66.05	45.87	23.82	49.51	42.05
Mean O %	8.60	13.50	18.86	16.69	15.78
Mean C %	11.21	29.42	40.79	24.61	26.20
Mean N %	14.14	11.21	16.53	9.19	13.12
Mean Al %					2.85

*spCPTi* smooth pickled titanium, *sehCPTi* hydrophilic smooth and acid-etched titanium, *CPTi Promote* titanium Promote, *LL CPTi* Laser-Lok titanium, *LL TAV* Laser-Lok titanium aluminium vanadium

### Scanning electron microscopy

**Fig. 3:**
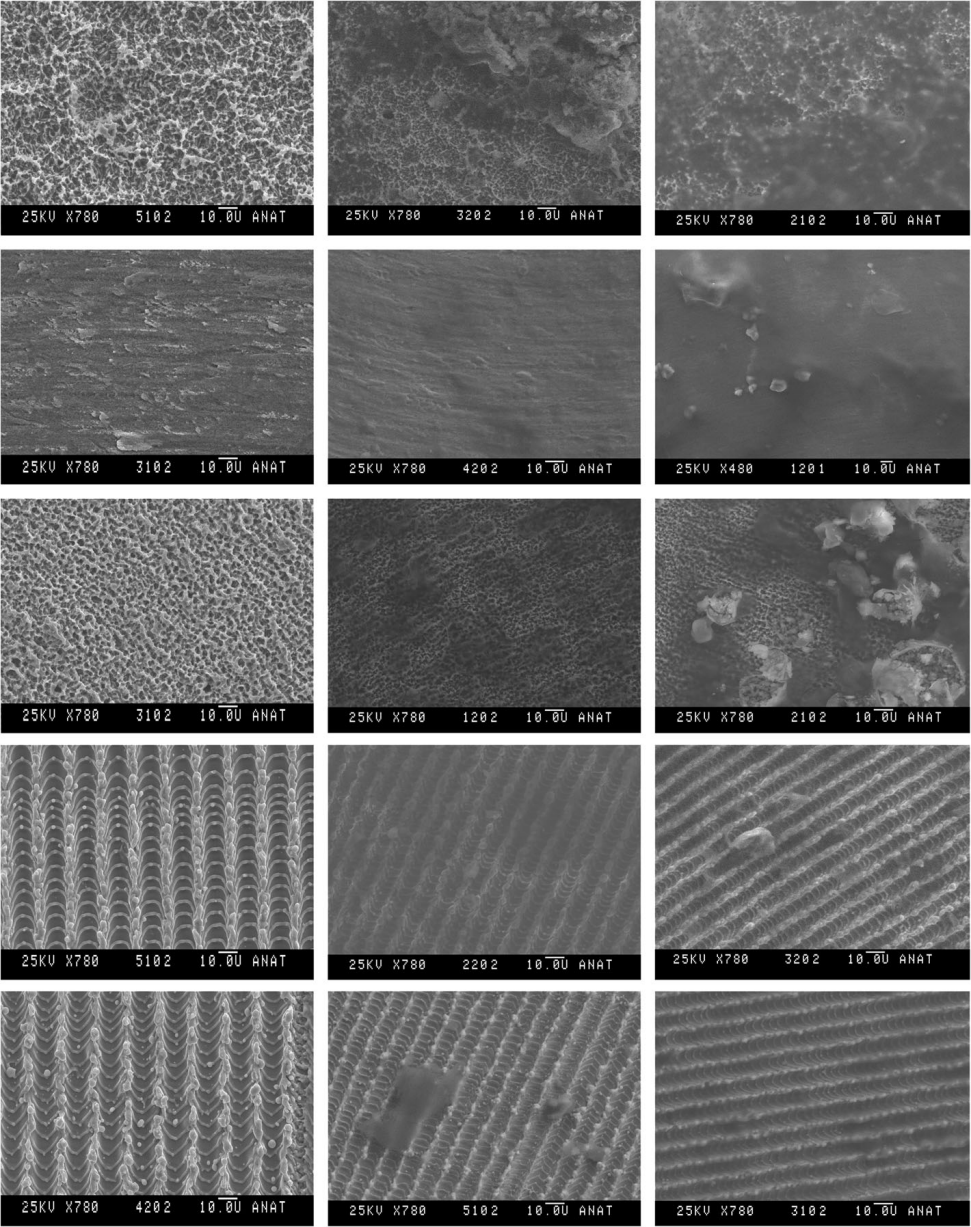
Images of scanning electron microscopy, showing different native samples on the left side, samples after 24 h and 48 h biofilm collection regarding the groups. spCPTi, smooth pickled titanium; sehCPTi, hydrophilic smooth and acid-etched titanium; CPTi Promote, titanium Promote; LL CPTi, Laser-Lok titanium; LL TAV, Laser-Lok titanium aluminium vanadium

Scanning electron microscopy confirmed the results of the quantitative biofilm analysis via erythrosine staining. Because of the covering of the different surfaces by developing biofilm, it was more difficult to focus on the surfaces by longer biofilm formation times. Especially on the smooth pickled and the promote surfaces, it was hardly possible to focus and depict the primary surfaces. On both Laser-Lok surfaces, only a thin layer of biofilm could be detected. Thus, the primary surfaces of these groups were still visible (Fig. 3).

## Discussion

In the contemporary study, the initial biofilm development on two Laser-Lok surfaces was compared to that one on other structured surfaces as well as on a smooth machined surface. The study design was chosen to mimic the natural biofilm development on implant surfaces. In contrast to other studies, cultivating artificial biofilms with several bacterial strains under laboratory conditions [11–14] biofilm was collected intraorally in the current study, which is closer to the situation in the oral cavity of the patients. It is known that the adhesion of in vivo biofilm to surfaces is higher compared to in vitro conditions [15]. Therefore, an established splint design was used, which was described in several studies before [16–19]. Undisturbed biofilm formation was guaranteed via turning the implant surfaces to the palate in a distance of nearly 1 mm. In this way, a moist and nutritious environment was ensured without the disturbing side effects of tongue or cheek movements. In this manner, the biofilm collecting time could be shortened for minimizing the risk of adverse health effects. Another aspect for choosing this short timeframe of biofilm formation lays in the fact that the study investigated initial biofilm formation quantitatively and not qualitatively. The time slot of 48 h for biofilm formation is the best, since studies showed a complete or almost complete coverage of different samples after 48 h of intraoral biofilm formation [17–23]. If longer periods of biofilm formation are determined, no differences between the various surfaces will be detected.

In the current study, both microtextured titanium Laser-Lok surfaces showed significant less and slower initial plaque area than the other tested surfaces. Laser-Lok titanium aluminium vanadium surface (42.8%) and Laser-Lok titanium (52.8%) showed significant less initial plaque areas compared to all control groups of the current study and depicted comparable results regarding mean initial plaque areas after 24 h of intraoral exposure to hydrophobic sandblasted large grid and acid-etched titanium and zirconium surfaces (48.7%), machined, acid-etched and chemically modified titanium and zirconium surfaces (40.6%) as well as super hydrophilic machined, acid-etched and chemically modified titanium surfaces (38.6%) in a similar designed investigations [20]. After 48 h of intraoral exposure Laser-Lok titanium aluminium vanadium (80.1%) and Laser-Lok titanium (83.4%), surfaces showed significant less initial plaque area than all control groups of the current study and even less initial plaque area compared to hydrophobic sandblasted large grid and acid-etched titanium and zirconium surfaces (98.9%), machined, acid-etched and chemically modified titanium and zirconium surfaces (95.6%) as well as super hydrophilic machined, acid-etched and chemically modified titanium surfaces (94.6%) of alike conceived examinations [20]. Interestingly, the hydrophobic Laser-Lok surfaces showed less initial biofilm formation after 24 as well as after 48 h intraoral exposition of samples than the hydrophilic machined, acid-etched and chemically modified titanium surface, which is in contrast to other previous investigations, mostly showing slower initial biofilm formation on hydrophilic implant surfaces [17, 20]. This could describe an exceptional position of laser-microtextured implant surfaces. Even after 48 hours both Laser-Lok surfaces were not completely covered by biofilm while most surfaces are almost completely covered by plaque after 48 hours [17–23]. In congruence to earlier examinations was the finding that machined surfaces showed higher biofilm accumulation than modified surfaces [17, 21]. This refutes again the former claim that roughness of implant surfaces has a higher impact on initial biofilm formation than other factors [24–26].

Another important issue especially regarding the implant maintenance is the cleanability of implant surfaces to prevent peri-implant infections. In the current investigation, a professional decontamination procedure was also tested as a domestic hygiene procedure via tooth brushing. After using an air abrasive device with glycine powder blasting on all surfaces nearly complete clean implant surface could be achieved, ranging from 99.71 to 99.94%. This is in accordance to earlier studies confirming the high effectivity of air abrasive device on smooth or rough/structured implant surfaces [18, 19, 27] proving the high effectiveness of air abrasive device. The greater challenge is the biofilm removal via tooth brushing. In the current investigation the highest clean implant could be detected on machined surfaces (98.8%), which also could be found in previous examinations [20]. On structured implant surfaces, usually more biofilm remnants can be found after tooth brushing, ranging from 69.5 to 39.4% [28]. Both Laser-Lok surfaces in the contemporary study showed 67.2 to 69.8% clean implant surface, which is a quite high level after tooth brushing. Maybe the parallel rims with their smooth bottoms, being the soonest comparable to machined surfaces, are easier to clean. Treatment time was also investigated in this study and not defined. If treatment time is defined too long, no differences regarding clean implant surface will be detected because in the worst case, all surfaces will show 100% clean implant surface. Even if one decontamination method is as effective as mentioned above, it is also valuable to know facts about the efficiency. If treatment time is fixed, important information will be lost. That is why the current study investigated treatment time as described above. The short treatment times of the air abrasive device groups verified the high efficiency of the air abrasive device as described previously [18, 19]. The treatment times of the tooth brushing groups on rough surfaces in the current study are longer than that ones described by Koh et al [28]. Maybe this could be an explanation of the higher clean implant surface level. But it also should be annotated that Koh et al. used one bacterial strain, porphyromonas gingivalis, which was incubated for two to three days on the samples, while in the current study intraoral biofilm was collected for two days, which is more complex than an artificial cultured bacterial lawn and should be more difficult to remove. For interpretation of these findings, it is important to know, that because of ethical reasons only probands with good oral hygiene and healthy periodontal conditions were included in this study. The composition of the biofilm is not comparable to that one of patients with periodontal or peri-implant infections. Regarding biocompatibility, it was observed that on the native samples significant higher cell viabilities could be detected compared to the decontaminated samples. Interestingly, the cell viabilities of both Laser-Lok groups were the highest ones observed in the current study, without any significance but by tendency. Once again it could be determined that the titanium surfaces once covered by biofilm could not be rehabilitated regarding biocompatibility only by removal of the biofilm as proved by former studies [18, 19]. Furthermore, highest cell viability could be detected on machined surfaces again [20]. Maybe this tendency is again correlated to the higher clean implant surface rates on the samples within both treatment groups. Electron dispersive X-ray analysis proved the apparent influence of the biofilm to the titanium surfaces. In every case, the titanium rate decreased, while the rate of carbon and sodium increased after covering by biofilm, even in the groups with high clean implant surface rates after treatment procedures. Contamination by biofilm, even initial biofilm, definitely seems to influence the composition of the titanium surfaces, as observed in previous studies [18–20].

## Conclusion

Within the limitations of this study, it can be concluded that both Laser-Lok surfaces showed slower and less initial biofilm formation for 24 or 48 h compared to smooth pickled titanium surface, hydrophilic smooth and acid-etched titanium and Promote surfaces. The smooth pickled titanium surface seemed to have advantages regarding decontamination procedures resulting in a higher clean implant surface, especially after tooth brushing. Both Laser-Lok surfaces showed a higher clean implant surface after decontamination procedures in comparison to hydrophilic smooth and acid-etched titanium surface and Promote surface. Because of this fact in combination with the slower biofilm formation on the Laser-Lok surfaces, these surfaces seem to be interesting candidates for the use in transmucosal components in implant designs. The superior biocompatibility of both Laser-Lok surfaces affirms this assumption. Of course, the results of the current study have to be interpreted carefully since this study depicts an ex vivo model. More studies are needed to examine these tendencies.

## Data Availability

All data generated or analysed during this study are included in this published article [and its supplementary information files].

## Abbreviations

LLCPTi: Laser-microtextured titanium surfaces
LLTAV: Laser-microtextured titanium aluminium vanadium surfaces
spCPTi: Smooth pickled titanium surfaces
sehCPTi: Hydrophilic smooth and acid etched titanium surfaces
CPTi Promote: Sandblasted large grid and acid etched titanium surfaces
CIS: Clean implant surface
IPA: Initial plaque area
LL: Laser-Lok surfaces
CPS: Counts per second
HGF: Human gingival fibroblasts
PI: Plaque Index

## References

[1] Lindhe J, Meyle J (2008). Peri-implant diseases: Consensus report of the sixth European Workshop on Periodontology. J Clin Periodontol.

[2] Quirynen M, De Soete M, van Steenberghe D (2002). Infectious risks for oral implants: a review of the literature. Clin Oral Implants Res.

[3] Fürst M, Salvi GE, Lang NP, Persson GR (2007). Bacterial colonization immediately after installation on oral titanium implants. Clin Oral Implants Res.

[4] Salvi GE, Fürst MM, Lang NP, Persson GR (2008). One-year bacterial colonization patterns of Staphylococcus aureus and other bacteria at implants and adjacent teeth. Clin Oral Implants Res.

[5] Esposito M, Grusovin MG, Tzanetea E, Piatelli A, Worthington HV (2010). Interventions for replacing missing teeth: treatment of periimplantitis. Cochrane Database Syst Rev.

[6] Shapoff CA, Lahey B, Wasserlauf PA, Kim DM (2010). Radiographic analysis of crestal bone levels around Laser-Lok collar dental implants. Int J Periodontics Restorative Dent..

[7] Farronato D, Mangano F, Briguglio F, Iorio-Siciliano V, Riccitiello F, Guarnieri R (2014). Influence of Laser-Lok surface on immediate functional loading of implants in single-tooth replacement: a 2-year prospective clinical study. Int J Periodontics Restorative Dent..

[8] Gopalakrishnan D, Joshi V, Romanos GE (2014). Soft and hard tissue changes around laser microtexture single tooth implants--a clinical and radiographic evaluation. Implant Dent..

[9] Iorio-Siciliano V, Marzo G, Blasi A, Cafiero C, Mignogna M, Nicolò M (2014). Soft and hard tissue modifications at immediate transmucosal implants (with Laser-Lok microtextured collar) placed into fresh extraction sites: a 6-month prospective study with surgical reentry. Int J Periodontics Restorative Dent..

[10] Guarnieri R, Grande M, Ippoliti S, Iorio-Siciliano V, Riccitiello F, Farronato D (2015). Influence of a Laser-Lok surface on immediate functional loading of implants in single-tooth replacement: three-year results of a prospective randomized clinical study on soft tissue response and esthetics. Int J Periodontics Restorative Dent..

[11] Socransky SS, Haffajee AD, Cugini MA, Smith C, Kent RL (1998). Microbial complexes in subgingival plaque. J Clin Periodontol.

[12] Socransky SS, Haffajee AD (2005). Periodontal microbial ecology. Periodontol 2000.

[13] Wiedmer D, Petersen FC, Lönn-Stensrud J, Tiainen H (2017). Antibacterial effect of hydrogen peroxide-titanium dioxide suspensions in the decontamination of rough titanium surfaces. Biofouling..

[14] Eick S, Meier I, Spoerlé F, Bender P, Aoki A, Izumi Y, Salvi GE, Sculean A (2017). In vitro-activity of Er:YAG laser in comparison with other treatment modalities on biofilm ablation from implant and tooth surfaces. PLoS One.

[15] Quirynen M, Marechal M, Busscher HJ, Weerkamp AH, Arends J, Darius PL, van Steenberghe D (1989). The influence of surface free energy on planimetric plaque growth in man. J Dental Res.

[16] John G, Becker J, Schwarz F (2013). Rotating titanium brush for plaque removal from rough titanium surfaces - an in vitro study. Clin Oral Implants Res.

[17] John G, Becker J, Schwarz F (2015). Modified implant surface with slower and less initial biofilm formation. Clin Implant Dent Relat Res.

[18] John G, Schwarz F, Becker J (2015). Taurolidine as an effective and biocompatible additive for plaque-removing techniques on implant surfaces. Clin Oral Investig.

[19] John G, Becker J, Schwarz F (2016). Effectivity of air-abrasive powder based on glycine and tricalcium phosphate in removal of initial biofilm on titanium and zirconium oxide surfaces in an ex vivo model. Clin Oral Investig..

[20] John G, Becker J, Schwarz F (2017). Effects of different titanium zirconium implant surfaces on initial supragingival plaque formation. Clin Oral Implants Res..

[21] Schwarz F, Sculean A, Wieland M, Horn N, Nuesry E, Bube C, Becker J (2007). Effects of hydrophilicity and microtopography of titanium implant surfaces on initial supragingival plaque biofilm formation. A pilot study. Mund Kiefer Gesichtschir.

[22] Siegrist BE, Brecx MC, Gusberti FA, Joss A, Lang NP (1991). In vivo early human dental plaque formation on different supporting substances. A scanning electron microscopic and bacteriological study. Clin Oral Implants Res.

[23] Rimondini L, Fare S, Brambilla E, Felloni A, Consonni C, Brossa F, Carrassi A (1997). The effect of surface roughness on early in vivo plaque colonization on titanium. J Periodontol.

[24] Quirynen M, Marechal M, Busscher HJ, Weerkamp AH, Darius PL, van Steenberghe D (1990). The influence of surface free energy and surface roughness on early plaque formation. An in vivo study in man. J Clin Periodontol.

[25] Amoroso PF, Adams RJ, Waters MG, Williams DW (2006). (2006). Titanium surface modification and its effect on the adherence of Porphyromonas gingivalis: an in vitro study. Clin Oral Implants Res.

[26] Teughels W, Van Assche N, Sliepen I, Quirynen M (2006). Effect of material characteristics and/or surface topography on biofilm development. Clin Oral Implants Res.

[27] Schwarz F, Ferrari D, Popovski K, Hartig B, Becker J (2009). Influence of different air-abrasive powders on cell viability at biologically contaminated titanium dental implants surfaces. J Biomed Mater Res B Appl Biomater.

[28] Koh M, Park JB, Jang YJ, Ko Y (2013). The effect of pretreating resorbable blast media titanium discs with an ultrasonic scaler or toothbrush on the bacterial removal efficiency of brushing. J Periodontal Implant Sci.

